# Using RNA-Seq to assemble a rose transcriptome with more than 13,000 full-length expressed genes and to develop the WagRhSNP 68k Axiom SNP array for rose (*Rosa* L.)

**DOI:** 10.3389/fpls.2015.00249

**Published:** 2015-04-21

**Authors:** Carole F. S. Koning-Boucoiran, G. Danny Esselink, Mirjana Vukosavljev, Wendy P. C. van 't Westende, Virginia W. Gitonga, Frans A. Krens, Roeland E. Voorrips, W. Eric van de Weg, Dietmar Schulz, Thomas Debener, Chris Maliepaard, Paul Arens, Marinus J. M. Smulders

**Affiliations:** ^1^Wageningen UR Plant Breeding, Wageningen University and Research CentreWageningen, Netherlands; ^2^Abteilung Molekulare Pflanzenzüchtung, Institute for Plant Genetics, Leibnitz University HannoverHannover, Germany

**Keywords:** *Rosa*, transcriptomics, EST, SNP array, genotyping, assembly

## Abstract

In order to develop a versatile and large SNP array for rose, we set out to mine ESTs from diverse sets of rose germplasm. For this RNA-Seq libraries containing about 700 million reads were generated from tetraploid cut and garden roses using Illumina paired-end sequencing, and from diploid *Rosa multiflora* using 454 sequencing. Separate *de novo* assemblies were performed in order to identify single nucleotide polymorphisms (SNPs) within and between rose varieties. SNPs among tetraploid roses were selected for constructing a genotyping array that can be employed for genetic mapping and marker-trait association discovery in breeding programs based on tetraploid germplasm, both from cut roses and from garden roses. In total 68,893 SNPs were included on the WagRhSNP Axiom array. Next, an orthology-guided assembly was performed for the construction of a non-redundant rose transcriptome database. A total of 21,740 transcripts had significant hits with orthologous genes in the strawberry (*Fragaria vesca* L.) genome. Of these 13,390 appeared to contain the full-length coding regions. This newly established transcriptome resource adds considerably to the currently available sequence resources for the *Rosaceae* family in general and the genus *Rosa* in particular.

## Introduction

Whereas, cut rose is economically the most important ornamental crop worldwide (761 million euros in The Netherlands in 2011), the rose genome sequence has not been completed yet. In fact, a long history of interspecific hybridization and selection (Debener and Linde, 2009; Smulders et al., 2011; Vukosavljev et al., 2013; Zhang et al., 2013) has led to a complicated taxonomy that is not fully resolved (Koopman et al., 2008; Fougère-Danezan et al., 2015). Commercial rose cultivars are mostly tetraploid and highly heterozygous. Therefore, inheritance patterns of quantitative traits may be complex. Phenotypic traits such as flower stem production, flower shape, flower color or disease resistance are of economic importance for breeders and growers (Debener and Linde, 2009; Smulders et al., 2011), and need to be better understood genetically in order to be able to apply marker-assisted selection in breeding programs. There is a consensus genetic map for rose (Spiller et al., 2011).

EST sequences are an efficient source of various markers for the construction of dense genetic linkage maps and the identification of QTLs (Vukosavljev et al., 2015). Based on next-generation sequencing technologies, four EST studies on roses have been published so far. Dubois et al. (2012) produced ESTs from 13 rose tissues and studied the expression of genes involved in flowering and scent biosynthesis. Yan et al. (2014) analyzed gene expression during flower blooming, whereas Kim et al. (2012) focused on miRNAs related to color genes in four rose cultivars. Yan et al. (2015) analyzed ascorbate biosynthesis genes and transcription factors in *Rosa roxburghii* fruits. These studies used a *de novo* assembly pipeline. An orthology-guided reference transcriptome assembly was developed by Ruttink et al. (2013) in *Lolium*, in order to be able to improve the assembly sequences from a highly heterozygous species in the absence of a reference genome sequence. The Genome Database for Rosaceae (GDR, Jung et al., 2014) contains, besides about 500,000 rose ESTs (2013), data of the fully sequenced woodland strawberry (*Fragaria vesca* L.) genome (Shulaev et al., 2011) that may be used as a reference genome for comparative genomics in rose. Strawberry is the closest related species for which a genome sequence is available.

SNP array platforms have been developed for various *Rosaceae* crops including apple (Chagné et al., 2012; Bianco et al., 2014), cherry (Peace et al., 2012), and peach (Verde et al., 2012). They have been shown to be an important resource facilitating the production of dense genetic maps and subsequent QTL mapping of important traits, genome-wide association analysis, pedigree-based analysis, and genomic selection (Bianco et al., 2014). The required density depends on the type of application and on the degree of LD on the germplasm—which in outcrossing species is often small (a few centimorgan). Dense maps are necessary to be able to identify haplotypes and study patterns of introgression with sufficient resolution power (Zhang et al., 2013). Tightly linked markers improve the efficiency of marker-assisted selection in breeding (Jänsch et al., 2015). In order to generate a versatile and large SNP array for rose, we set out to mine ESTs from diverse sets of germplasm comprising tetraploid cut and garden roses as well as a diploid garden rose, so that the SNPs on the array would be polymorphic in a wide range of rose genetic backgrounds.

Here, we present the results of (i) three transcriptome *de novo* assemblies based on tetraploid cut and garden rose genotypes and a diploid rose, (ii) the development of a 68K genotyping SNP marker array on the Axiom platform, and the (iii) construction and (iv) annotation of a non-redundant rose transcriptome for tetraploid roses using the genome sequence of diploid strawberry. This study generates valuable genomic resources (i.e., EST library with annotations and genetic diversity between different genotypes) and resulted in the construction of a large SNP array that can serve as a genotyping platform for future studies in diploid and tetraploid roses.

## Materials and methods

### Plant material

**Figure 1:**
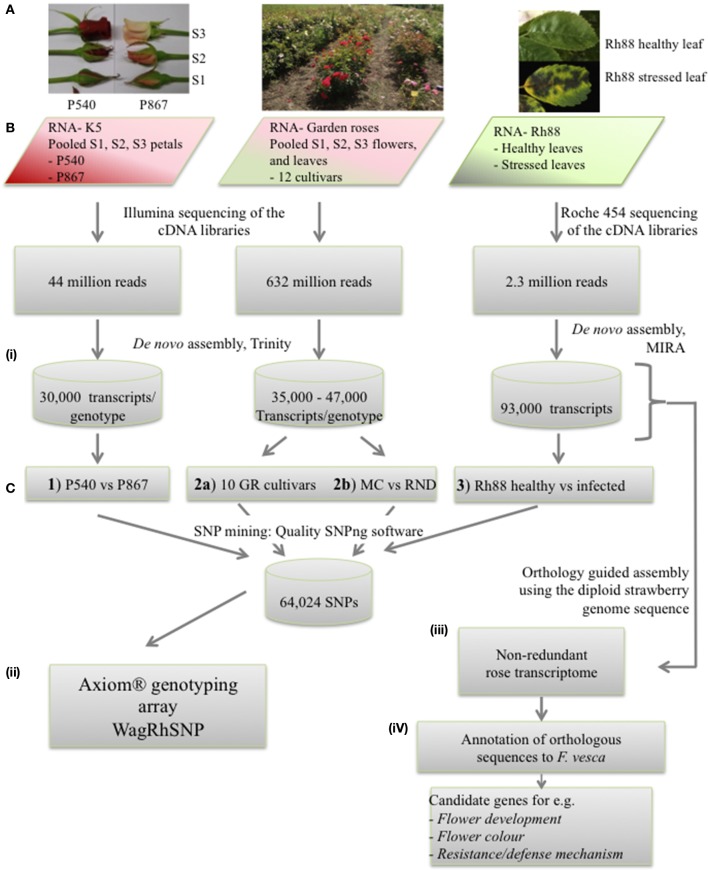
**Overview of the strategy used to assemble the rose EST data, to mine SNPs, and to develop the WagRhSNP Axion SNP array. (A)** Pictures of the plant material used to isolate RNA. **(B)** Sequencing of the three sets. **(C)** Data analysis: (i) SNP mining in the three sample sets (GR, garden roses; MC, Morden Centennial; RND, Red New Dawn), (ii) development of the WagRhSNP Axiom® genotyping array, (iii) transcriptome assembly, and (iv) identification and annotation of sequences orthologous with *F. vesca*. All SNPs are in Supplementary file ESM2. The SNPs identified in set 1) are coded with a ‘K’, those in 2a) with a ‘G’, in 2b) with a ‘M’ and in 3) with a ‘D’.

Three sources of material were used. From the parents of the K5 segregating population of tetraploid cut roses (*Rosa hybrida*, Yan et al., 2005; Koning-Boucoiran et al., 2012), P540 (mother) and P867 (father), petals were harvested in three stages of flower development (S1, S2, S3, Figure 1A). This material is designated as K5. For garden roses (designated as GR) whole flowers at three flowering stages (closed, half-opened, and fully-opened) as well as young leaves were harvested of 12 European and Canadian garden rose cultivars: Morden Fireglow, Adelaide Hoodless, Prairie Joy, Morden Blush, Diamond Border, Nipper, J.P. Connell, Princess of Wales, Heritage, Graham Thomas, Morden Centennial (MC), and Red New Dawn (RND) (Figure 1A; Vukosavljev et al., 2013). For the diploid *Rosa multiflora* hybrid 88/124-46 (Biber et al., 2010; designated as Rh88) leaves were harvested from plants grown under optimal growing conditions (control) and from plants 1–6 days after inoculation with black spot, *Diplocarpon rosae* (Debener et al., 1998), powdery mildew, *Podosphaera pannosa* (Linde and Debener, 2003), or downy mildew, *Peronospora sparsa* (Schulz et al., 2009), 1 h after wounding or after 1 h of 40°C heat stress. Sampled tissues were immediately frozen in liquid nitrogen and stored at −80°C until RNA extraction.

### RNA preparation

Total RNA was isolated from P540 and P867 by using the RNeasy Plant Mini Kit (QIAGEN, Westburg, The Netherlands) according to the manufacturer's instructions. The K5 RNA samples were prepared by pooling equal amounts of total RNA isolated from petals of the three flower stages as mentioned above. For GR total RNA was isolated from the 12 garden roses according to Chang et al. (1993) with modifications described in Supplementary Table ESM 1. Total RNA samples were prepared by pooling equal amounts of RNA from the four samples (three flower stages and leaves). For Rh88 total RNA from treated and untreated leaves was isolated using the Invitek RNA extraction kit (STRATEC Molecular GmbH, Berlin, Germany) according to the manufacturer's instructions. Remaining DNA was removed by digestion with RNAse-free DNAse as specified in the extraction kit.

### Sequencing of cDNA

RNA samples of the K5 parents were sequenced by ServiceXS (Leiden, The Netherlands) using Illumina's standard operation protocols (2 × 75 bp paired end) on a Genome Analyser II. The RNA samples from the 12 garden rose cultivars (GR) were sent to GATC Biotech (Constance, Germany) where 12 cDNA libraries were sequenced using 2 × 100 bp paired end sequencing on a HiSeq 2000. For the pools of stressed and unstressed Rh88 leaves two random primed normalized cDNA libraries were constructed and sequenced in two 454 FLX Titanium runs at the Roche 454 sequencing center in Branford (USA). The first pool consisted of eight independent RNA isolations of untreated leaves and the second pool consisted of equal amounts of RNA from four independent extractions of all stress-treated leaves.

### Pre-processing of the sequences

Illumina reads were pre-processed using Prinseq-lite (vs. 0.20.3) which included the trimming of nucleotides having a phred score lower than 25, the trimming of 10 nucleotides from the 5′ end to remove the bias of the nucleotide content of the reads due to the random hexamer priming (Hansen et al., 2010), the trimming of poly A/T tails, the removal of duplicate reads, of low complexity reads (DUST approach), of reads shorter than 50 nucleotides and of reads with more than one ambiguous nucleotide. Next, the paired-end reads were processed for overlapping sequences using COPE (Liu et al., 2012). All unconnected reads were used as normal paired-end reads, all connected read pairs (i.e., merged read pairs) and single reads were used as single-end reads.

The 454 reads were pre-processed using the FASTX toolkit (v0.0.13) with the same trimming and filtering as the Illumina reads except that reads smaller than 100 bp were discarded. Duplicate reads were removed using USEARCH v5.2.32 (Edgar, 2010). The reads were corrected for homopolymer nucleotide tracks using Acacia v1.52 (Bragg et al., 2012).

### Transcriptome *De novo* assembly

*De novo* transcriptomes were assembled per sample set using Trinity (min_kmer_cov 2, Grabherr et al., 2011) for the Illumina datasets for transcriptome assembly and SNP calling. For the 454 dataset the MIRA/CAP3 assemblers in the iAssembler (v1.3.2) pipeline (Zheng et al., 2011) were used for the transcriptome assembly, and the CLC assembler for SNP detection, in which sequences of the control and stressed leaves were combined to increase the number of reads. To select for relevant biological transcripts within each data set, RSEM (RNA-Seq by Expectation-Maximization) was used for transcript abundance estimation. If less than 1% of the total reads of a component (IsoPct) matched with a specific transcript, the transcript was not included in the subsequent steps of the analysis. Of the others the most abundant transcript/isoform was selected. To this end, all reads were mapped against all transcripts. Rh88 transcripts were also filtered for fungal sequences by blasting against available fungal sequences of *Marssonina brunnea* (Zhu et al., 2012).

### SNP mining

SNPs were identified within subsets of the sequences: (1) between K5 parents P540 and P867, (2a) among 12 garden rose cultivars, (2b) in a subset of those, namely between the two garden roses MC and RND (Vukosavljev et al., in preparation), and (3) within Rh88 (Figure 1C). For each of the sets the individual transcriptomes were assembled with CAP3 (default settings with–p 97) to generate a reference transcriptome per dataset. All reads of each dataset were mapped to their specific reference transcriptome with Bowtie 2 (Langmead and Salzberg, 2012) with modified settings (—very-sensitive—rfg 5, 10) and filtered for map quality (>25) using SAMtools.

The resulting SAM file was used for SNP calling using QualitySNPng (Nijveen et al., 2013) with modified settings (the minimal similarity score per polymorphic site, similarityAllPolymorphicSites: 0.8, the minimal number of reads per allele set at 5). Using the filtering options of QualitySNPng, SNPs found in transcripts displaying a larger number of haplotypes than theoretically expected were discarded. For the tetraploid parents of each mapping population (P540 × P867 and MC × RND), a maximum of 8 haplotypes (four haplotypes per parent) per transcript was expected. The resulting SNP markers with 35 flanking nucleotides on both sites without additional SNPs or InDels were marked as potential markers. To filter against paralogous markers, transcripts containing selected SNP markers were searched against their own reference transcripts (BLASTn *e*-value 1-30). Transcripts with two or more hits were discarded since this indicates that they may be present several times in the genome.

To prevent interference with chloroplast DNA during the array hybridization all sequences around SNP markers were screened against the chloroplast genome of *Fragaria vesca* (http://www.rosaceae.org). The genome sequence of *Fragaria vesca* was used to identify and remove sequences with potential splice junction sites. For this, markers were searched against the Fragaria sequences (BLASTn *e*-value 1-5) and discarded using custom perl scripts if their sequences matched with fewer than 68 bp (95%). All A/T and C/G polymorphisms were also excluded since genotyping these SNPs requires twice the number of probes using the Axiom platform. As a last step the mined SNP markers of the three sets were compared to remove redundancy across sets.

### Axiom genotyping array: WagRhSNP

The selected SNPs were submitted to Affymetrix (Santa Clara, CA, USA) for a final analysis to determine whether the probes could be synthesized. Affymetrix discarded SNPs that shared similar sequences, as this could interfere with hybridization. We decided to include two probes for each SNP on the array, each probe targeting one of the strands (coded as AX_set_ID), as this allows an additional quality check during genotype calling with dosage scoring (Smulders et al., 2015). The array also includes 3000 non-polymorphic control probes (coded as DQC-sample name, DQC is a measure of the extent to which the distribution of signal values is separated from background values). The SNPs on the array are listed in Supplementary Table ESM 2, mentioning for each SNP its SNP_ID, the flanking sequences, the alleles of the SNP, the Rh-Fv ortholog transcript code, the *Fragaria vesca* protein code, and the annotation in *Fragaria* whenever available.

### Orthology-guided reference transcriptome assembly

An orthology-guided assembly procedure according to Ruttink et al. (2013) was followed for the construction of a non-redundant rose transcriptome sequence. The set of non-redundant proteins of *Fragaria vesca* was downloaded from PLAZA2.5 (Van Bel et al., 2012) to guide the assembly. tBLASTn, carried out in 2013, (*e*-value cut-off 1e-5 and up to 250 hits allowed) was used to search for protein hits (*e*-value 1e-10) with all retained rose transcripts of this study. The transcripts with a significant tBLASTn hit with a *F. vesca* protein were grouped and assembled using CAP3 with default settings. The assembled transcripts were compared to the *F. vesca* proteins using BLASTx, and if the highest-scoring protein returned the *Fragaria* gene originally used as the highest scorer, then the two genes were considered as putative orthologs, and the transcript was selected and tentatively named after the most likely orthologous gene in *F. vesca* (Rh-Fv transcripts). Next, the longest ORF of each selected rose sequence was determined (Trinity package, Grabherr et al., 2011), the 3′ and 5′ UTR sequences trimmed off and the remaining sequences were reassembled using CAP3 with default settings to select the final set of orthologous sequences of rose.

Functional domains predicted in *Fragaria* and available in GDR (Jung et al., 2014; http://www.rosaceae.org) were mined for our rose Rh-Fv transcripts. They were also scanned for protein signatures from superfamilies reported in various databases such as Smart, Tigr, Panther, Pfam, FPrint, Profilescan, ProDom, and Gene3D (Zdobnov and Apweiler, 2001).

## Results

### Individual *De novo* transcriptome assemblies

RNA-Seq results of K5 (two cut rose genotypes) and GR (12 garden rose cultivars) were obtained by using Illumina paired-end sequencing (Figure 1A, Table 1). To generate the RNA-Seq data of Rh88 (garden rose Rh88 hybrid, Figure 1A) 454 sequencing was used.

**Table 1:**
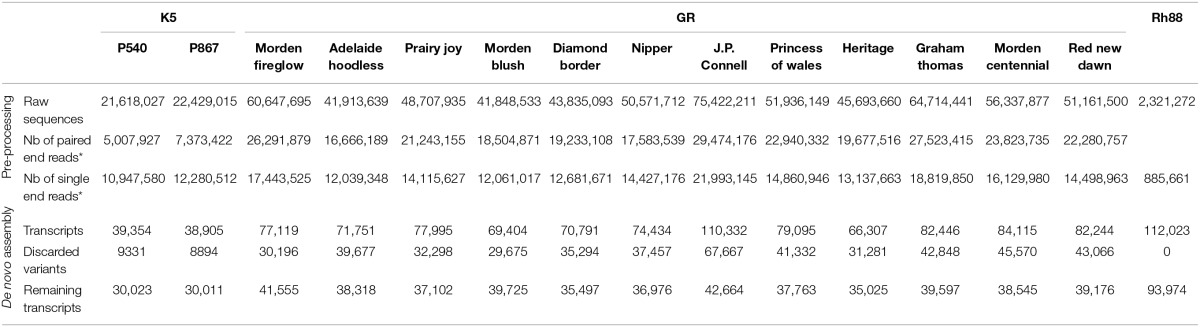
**Sequencing results and analysis**.

*Number of paired-end reads and single reads per sample after quality trimming and merging during pre-processing of the data.

Sequencing of cDNAs from petals of the tetraploid cut rose K5 parents gave 44 million 75 bp paired-end reads. After quality trimming and merging, 81% of the reads remained either as paired-end (12 million) or as single end reads (23 million). In total ~78,000 transcripts were obtained, but ~18,000 (24%) of these were subsequently discarded since they were less abundant and possibly splice variants. For SNP calling it is better to avoid these. On one hand they could represent paralogous genes, which would lead to nucleotide differences between paralogs rather than between alleles of the same locus. On the other hand, if they are from splice variants they may map in multiple contigs, which could mean that all of these would unnecessarily be excluded from the SNP calling. Ultimately, approximately 30,000 transcripts per genotype (Table 1) were identified.

Similarly, the sequencing of cDNAs from petals and leaves of GR resulted in 632 million 100 bp paired-end reads. After quality trimming and merging, 71% of the reads remained either as paired-end or single end reads. After filtering of the most abundant transcripts during the assembly, 48% were discarded as possible splice variants. Ultimately, between 35,000 and 47,000 transcripts per cultivar were identified (Table 1).

The sequences of Rh88 of healthy and stressed leaves were combined and resulted in 2.3 million reads with an average length of 360 bp. After quality trimming, duplicate removal, and homopolymer correction 38% remained. Filtering of the most abundant transcripts yielded 93,974 transcripts (Table 1). This large number was partly due to the presence, in the black spot and powdery mildew infected leaves, of fungal genes. The sequences were therefore blasted (blastx) against available fungal sequences of *M. brunnea* (Zhu et al., 2012), based on which 12,705 sequences with an average homology of 53.4% were discarded. The remainder was used in further analysis.

### Development of the WagRhSNP array

**Figure 2:**
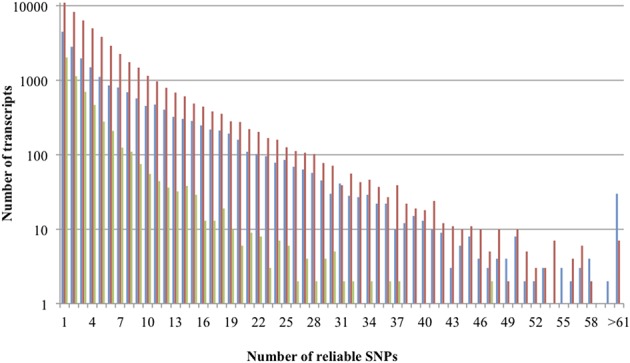
**Distribution of reliable SNPs per transcript of K5 (blue bars), GR (red bars), and Rh88 (green bars)**.

SNPs were mined in transcripts containing at least one reliable SNP (Tang et al., 2006) for the three rose datasets separately (Table 2). The smallest transcript was 135 bp long, assembled from 10 reads and showed 1 reliable SNP while the largest transcript was 14,270 bp long, from 15,286 reads, showing 77 reliable SNPs. Some transcripts contained up to 123 reliable SNPs. A small majority of the SNPs (62.3% for K5, 59.8% for GR, and 57.7% for Rh88) were transitions (C/T or G/A), as is common (in almond: 51%, Wu et al., 2008; in wheat and maize: 45% and 55% respectively, Edward et al., 2008; in cassava, up to 65%, Lopez et al., 2005). The average SNP density varied between 0.4 and 0.6 per 100 bp among the three sample sets but the variation between transcripts within each sample was large, as pointed out by the standard deviations (Table 2). The distribution of reliable SNPs per transcript is given in Figure 2 (note the log scale). Half of the transcripts had 1-3 SNPs per transcript.

**Table 2:** **Number of SNPs mined in the three rose datasets**.

**Sample set**	**Number of transcripts^a^**	**Average transcript length (bp)**	**STD**	**Average transcript coverage^b^**	**STD**	**Number of reliable SNPs per transcript**	**Average density SNP/100 bp**	**STD**
K5	19080	1134.5	952.2	643.0	2017.6	1–123	0.6	0.41
GR^c^	51106	1342.9	994.0	787.9	2700.3	1–96	0.5	0.40
Rh88	5493	1296.2	740.2	48.5	34.7	1–57	0.4	0.53

*STD = standard deviation*.

a *Transcripts containing at least one reliable SNP*.

b *Average of the number of reads per transcript*.

c *GR: All 12 garden rose cultivars included*.

The WagRhSNP array includes a total of 68,893 reliable SNPs. Of these, 26,354 SNPs were identified between the cut rose parents (labeled: K_transcript number_SNP number), 26,364 SNPs among the 12 garden rose cultivars (named: G_transcript number_SNP number), 14,293 SNPs between MC and RND (named: M_transcript number_SNP number) and 1882 SNPs between alleles of Rh88 (named D_transcript number_SNP number). Probes for both strands are on a genotyping array which we named the WagRhSNP rose array totaling 137,786 probes (described in Supplementary Table ESM 2). This Axiom®array is commercially available for genetic studies in rose. The availability of a signal from two independent probes for each SNP enables additional quality control during scoring of the signal dosage, which is important for accurate genotyping in tetraploids (Vukosavljev et al., in preparation; Arens et al., in preparation).

### Orthology-guided assembly of the rose transcriptome

The orthology-guided assembly procedure, developed for *Lolium* by Ruttink et al. (2013), was applied. The non-redundant protein coding sequences from strawberry (34,748 unigenes) were mapped against the 628,240 rose transcripts identified during the *de novo* assembly (Figure 1). In total 381,621 (60.7%) transcripts were mapped against 28437 strawberry unigenes. They could be assembled into 21740 orthologous sequences, whereby singletons (i.e., strawberry unigenes with only a hit against single rose transcript) were discarded. Of these 13,390 sequences (61.6%) corresponded to complete unique ORFs (Supplementary Table ESM 3).

A single *Fragaria* protein could map against up to 25 rose transcripts, which partly overlapped. For example, when looking at FV0G46670, the orthology-guided assembly identified 25 rose transcripts out of 250 transcripts that had been identified during the *de novo* assembly.

### Annotation of the rose transcripts

**Figure 3:**
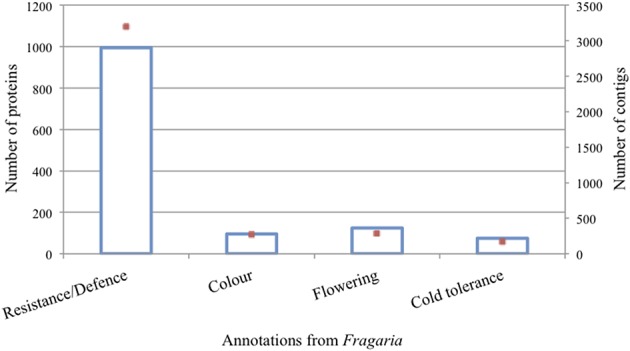
**Number of proteins (blue bars) and their respective transcripts (red dots) in which they were identified for relevant protein functions in the sampled tissues**.

The annotated rose transcripts with orthologs in *Fragaria* (named Rh-Fv transcripts) were investigated for functions and involvement in processes such as disease resistance and defense mechanisms, flower development and flower color (Supplementary Table ESM 4). The transcripts were further grouped into GO classes and functional domains, and were mined based on the InterPro Scan prediction of *Fragaria* (Zdobnov and Apweiler, 2001). A total of 2498 different protein domains were predicted to be present (Supplementary Table ESM 5). The putative annotations of the *Fragaria vesca* genome were linked to the protein domains identified in our dataset. Within those domains, 8090 proteins were identified based on 17,726 transcripts (Supplementary Table ESM 5). Figure 3 illustrates the distribution of four protein functions (resistance/defense, flower color, flowering, and cold tolerance) and the number of rose candidate genes identified within each functional class. For instance, 300,000 transcripts were annotated as involved in resistance/defense mechanisms, with homology to 1000 Fragaria proteins. Among them six different putative mlo-like genes (up to 25 transcripts) were identified out of the eight mlo-like genes present in the *Fragaria* genome. These included the full sequence of RhMLO1, RhMLO3 and RhMLO4, and a partial sequence of RhMLO2 (Kaufmann et al., 2012). The unique putative TMV resistance protein N matched with 270 rose genes.

GO terms were assigned to the annotated transcripts (Supplementary Table ESM 4). Around 20,000 genes (38%) were assigned to both molecular functions (such as transport, signal transduction, and structural molecules) and to biological processes (such as flower development, protein metabolism and response to stress). Around 10,000 genes (21%) were assigned to the category cellular compounds (e.g., organelle synthesis/regulation). Table 3 shows, for instance, that 978 transcripts belong to the GO class (GO:0004674) protein serine/threonine kinase activity, which includes not only the above identified protein (LRR receptor-like serine/threonine-protein kinase) but also other types of kinases involved in other processes (Supplementary Table ESM 4).

**Table 3:** **List of the top 20 gene ontology (GO) terms the most represented among the annotated transcripts from cut and garden roses, for each of three main GO categories**.

**GO terms**		**Number of transcripts**	**GO categories**
GO:0005515	Protein binding	1871	Molecular function 38.2%
GO:0003677	DNA binding	1077	
GO:0004674	Protein serine/threonine kinase activity	978	
GO:0003676	Nucleic acid binding	881	
GO:0003700	Transcription factor activity	828	
GO:0005488	Binding	613	
GO:0003824	Catalytic activity	588	
GO:0016491	Oxidoreductase activity	553	
GO:0003723	RNA binding	532	
GO:0005524	ATP binding	464	
GO:0000166	Nucleotide binding	410	
GO:0009055	RNA binding Electron carrier activity	376	
GO:0043565	Sequence-specific DNA binding	316	
GO:0004888	Transmembrane receptor activity	265	
GO:0016301	Kinase activity	262	
GO:0004497	Monooxygenase activity	251	
GO:0016758	Transferase activity	242	
GO:0020037	Heme binding	241	
GO:0004553	Hydrolase activity	218	
GO:0005215	Transporter activity	205	
GO:0006468	Protein amino acid phosphorylation	1027	Biological process 38.3%
GO:0055114	Oxidation reduction	1012	
GO:0006355	Regulation of transcription, DNA-dependent	644	
GO:0008152	Metabolic process	553	
GO:0055085	Transmembrane transport	534	
GO:0006508	Proteolysis	466	
GO:0006915	Apoptosis	423	
GO:0007165	Signal transduction	396	
GO:0006952	Defense response	340	
GO:0005975	Carbohydrate metabolic process	336	
GO:0006412	Translation	329	
GO:0045087	Innate immune response	260	
GO:0006457	Protein folding	258	
GO:0009651	Response to salt stress	244	
GO:0046686	Response to cadmium ion	232	
GO:0006629	Lipid metabolic process	186	
GO:0009793	Embryo development ending in seed dormancy	176	
GO:0009737	Response to abscisic acid stimulus	170	
GO:0006886	Intracellular protein transport	165	
GO:0006810	Transport	162	
GO:0005634	Nucleus	1401	Cellular compound 20.8%
GO:0016020	Membrane	1175	
GO:0005886	Plasma membrane	1172	
GO:0016021	Integral to membrane	634	
GO:0009507	Chloroplast	566	
GO:0005737	Cytoplasm	462	
GO:0005622	Intracellular	452	
GO:0005739	Mitochondrion	333	
GO:0005773	Vacuole	306	
GO:0005840	Ribosome	281	
GO:0031224	Intrinsic to membrane	259	
GO:0005829	Cytosol	243	
GO:0005783	Endoplasmic reticulum	205	
GO:0009941	Chloroplast envelope	199	
GO:0009570	Chloroplast stroma	188	
GO:0005618	Cell wall	177	
GO:0009505	Plant-type cell wall	173	
GO:0005730	Nucleolus	112	
GO:0005794	Golgi apparatus	98	
GO:0009535	Chloroplast thylakoid membrane	96	
			Others 3%

The Rh-Fv transcripts (i.e., those with orthologous sequences in the *Fragaria* genome) of our dataset were compared to the ROSAseq database (Dubois et al., 2012; http://iant.toulouse.inra.fr/R.chinensis). This database contains 80,714 rose EST clusters (based on 454 sequencing) longer than 100 nucleotides (average length of 444 ± 209.4 bp), annotated with the *Fragaria vesca* genome. Of these, 56,899 EST clusters had a BLASTn hit to 14,302 Rh-Fv transcripts of our study with a mean nucleotide identity of 96.2%. In general multiple ROSAseq EST clusters mapped to a single Rh-Fv transcript: 95% of these Rh-Fv transcripts matched with up to 10 EST clusters from the ROSAseq database, while ca. 85% of the ROSAseq EST clusters matched a single Rh-Fv transcript (Supplementary Table ESM 6). For instance, three not annotated ROSAseq EST clusters (RC013751, RC050162, and RC061808) were similar to Rh-Fv transcript FV1G02570.m1, which was annotated as a putative mlo-like protein 1. Furthermore, five EST clusters from the ROSAseq database (RC016326, RC022993, RC028093, RC040307, RC072319), four of which not annotated, showed similarity to one Rh-Fv transcript (FV1G02570.m1) annotated as putative mlo-like protein 6. On the other hand, ROSAseq clusters annotated as putative mlo-like proteins 2, 11, and 14 did not have any similarities with the Rh-Fv transcripts.

## Discussion

In this study, three sets of transcript sequence data were combined and analyzed in order to develop a SNP genotyping array. The generated markers may be used to produce dense genetic linkage maps at tetraploid and diploid level, improve QTL and gene function analyses, and the study of synteny with *Fragaria*. The genotyping array with 68,893 SNPs is commercially available for the *Rosaceae* community.

### The WagRhSNP axiom array

We chose to use the Axiom® array system of Affymetrix (Santa Clara, CA, USA). Advantages of the Axiom array system for SNP detection include the large number of probes that fit on the array, the small size of conserved probe sequences (so that additional SNPs do not interfere so often in sequences with a high density of SNPs), and that the array can be produced for 480 samples (5 microtiter plates) onwards using photolithographic templates (so that arrays ordered later will be identical to the original ones). Axiom arrays are being developed for other rosaceous crops as well, notably a 90K array for octoploid strawberry within the RosBREED project (Smulders et al., 2015).

By analyzing three data sets with two different sequencing platforms, more than 68k SNPs were identified and included on the WagRhSNP Axiom array. Per transcript the SNP frequencies of the K5 and the GR samples were 1 SNP every 167 bp and 200 bp, respectively, which is higher than the SNP frequency of 1 SNP/288 bp found in the highly heterozygous genome of apple (Chagné et al., 2012). SNPs on this array originate for approximately 40% from tetraploid cut roses and 60% from tetraploid garden roses, but it can be expected that they will be useful in all tetraploid germplasm, as cut roses represent a subset of the germplasm of garden roses (Vukosavljev et al., 2013). We included around 1000 SNPs identified in diploid *R. multiflora*. As tetraploid roses are the result of extensive hybridisation between (diploid) species and are probably segmental allopolyploids, many of the SNPs on the array that have been identified as polymorphism within and between tetraploid cultivars, may be polymorphic in diploid germplasm as well. Zhang et al. (2013) observed that SNP haplotypes at a SNPSTR locus were shared between tetraploid and diploid *Rosa* species.

The array will also be very useful as the SNPs reside in coding regions of genes that are being expressed. Genetic map positions of the SNP markers can thus be linked to transcript sequences and, if available, gene annotation. The Supplementary files contain the keys for the connections to the genes predicted in the *Fragaria vesca* genome sequence. Similarly, gene annotations can be screened for candidate genes, which then can be examined for the presence of SNPs.

### Assembly issues

The individual assemblies were performed without a reference genome and are therefore difficult to validate but they highlighted the diversity among the samples and they were used to identify reliable SNPs. Ruttink et al. (2013) indicated that *de novo* assemblies in highly heterozygous species typically yield more transcripts than the actual number of genes expressed, and that was the case here as well (Table 1). We proceeded to construct a common transcriptome from these samples by using orthology-guided assembly from Ruttink et al. (2013). The three different rose datasets had been produced with two different sequencing platforms (Illumina paired-end on a GAII and a HiSeq, and 454). The Illumina paired-end reads were short (110–200 bp) but we obtained up to 15,000 reads per transcript. The 454 reads of the diploid *R. multiflora* cultivar were longer (350 bp) but transcript depth was limited (max. 400 reads/transcript). Finseth and Harrison (2014) concluded that using Illumina reads alone one can produce a high quality transcriptome appropriate for RNA-Seq gene expression analyses, but that utilizing both 454 and Illumina is preferred. Hodgins et al. (2014) came to a similar conclusion.

### Annotation

The orthology-guided assembly based on the *Fragaria* genome (Supplementary Table ESM 3) produced 21,740 orthologs, of which 13,390 appeared to be full-length coding regions with an average length of 1089 bp. In this way, we could identify over 1/3 of all the genes estimated to be present in the rose genome, a good result as tissues such as root, stem, fruit, and seed were not included in this study. They provide an additional resource to the 14,252 peptides (EST clusters with an average length of 444 bp) orthologous to *Fragaria* identified by Dubois et al. (2012) in their ROSAseq database, which was produced from diploid roses.

## Conclusion

Our data provides the most comprehensive transcriptome resource currently available for rose with 13,390 expressed full-length genes identified. This resource adds significantly to the currently available genomics and bioinformatics resources for the genus *Rosa*. SNPs in many of these genes are present on the 68k WagRhSNP Axiom array, which will support candidate gene identification. The dense SNP array with 68,893 SNPs will enable producing dense genetic maps that are useful in genetic research and marker-assisted breeding.

## Author and contributions

Conception of the study: CK-B, VG, FK, WW, TD, CM, PA, MS; Collection of material: CK-B, MV, WW, VG, DS, PA; Production of the data: CK-B, MV, WW, DS, DT, MS; Assembly: DE; SNP selection and array design: GE, RV, WW, CM, PA, MS; Annotation of the rose transcripts: CK-B, GE; Writing of the manuscript: CK-B, PA and MS. All authors read and approved the final version of the manuscript.

### Conflict of interest statement

The authors declare that the research was conducted in the absence of any commercial or financial relationships that could be construed as a potential conflict of interest.

## Supplementary material

Supplementary Table ESM 1 **Adjusted protocol for RNA extraction from rose flowers based on Chang et al. (1993)**.(docx)

Supplementary Table ESM 2 **The complete description of all SNPs on the WagRhSNP array**. For each SNP the following is listed: SNP_ID, flanking sequences, and alleles of the SNP (A/B). In addition, the Rh-Fv ortholog transcript code, the *Fragaria vesca* protein code, and the annotation in *Fragaria* are given whenever available. This file provides all sequence information about the SNPs on the array and their flanking regions, but it can also be used as a key to the most similar sequence in the Fragaria vesca (Fv) genome, if available, and vice versa. (csv)

Supplementary Table ESM 3 **Orthologous ORF sequences of roses, constructed using the orthology-guided assembly procedure**. The sequence header contains the Rh_Fv ortholog name and the related Fragaria (Fv) transcript annotation. (csv) Supplementary Table 3 will be uploaded to GDR and ROSAseq.

Supplementary Table ESM 4 **InterPro predictions for the orthologous transcripts of cut and garden roses**. The predictions originate from the related Fragaria (Fv) transcript. (csv)

Supplementary Table ESM 5 **GO classes and GO annotations of the Rh-Fv transcripts**. The annotations originate from the related Fragaria (Fv) transcript. (csv)

Supplementary Table ESM 6 **Key from ROSASeq transcripts to Rh_Fv_orthologs**. The file contains ROSASeq transcripts with high similarity with Rh_Fv orthologous ORF sequences. The annotations originate from the related Fragaria (Fv) transcript. (csv)
