# Supplementary material for: Using RNA-Seq to assemble a rose transcriptome with more than 13,000 full-length expressed genes and to develop the WagRhSNP 68k Axiom SNP array for rose (Rosa L.)
Source: Front Plant Sci. 2015 Apr 21;6:249. doi: 10.3389/fpls.2015.00249 (PMC4404716; doi:10.3389/fpls.2015.00249)
Supplement: Supplementary Table ESM 1 — Adjusted protocol for RNA extraction from rose flowers based on Chang et al. (1993).(docx) [file Table1.DOCX]

ESM1

**Adjusted protocol for RNA extraction from rose flowers based on Chang et al. (1993).**

1. Grind the flowers in a mortar with liquid nitrogen. Keep the samples frozen in liquid nitrogen.
2. Weigh 1 to 1,5 g of material in a pre-cooled Greiner blue cap tube (50 ml)
3. Transfer 7.5 ml extraction buffer and 150 µl β-Mercaptoethanol to a 50 ml Greiner Blue cap tube.
4. Warm the tubes containing the extraction buffer and β-Mercaptoethanol to 65°C in a water bath.
5. Quickly add 1 to 1,5 g ground tissue and mix by inverting the tube.
6. Add 7.5 ml Chloroform:IAA (24:1) and mix vigorously.
7. Centrifuge the tubes in the Heraeus Multifuge3 S-R (Thermo Scientific) at 4600 rpm for 20 minutes at room temperature.
8. Transfer the supernatant (ca.7.5 ml) to a new Greiner blue cap tube (50 ml), add 7.5 ml Chloroform: IAA and mix vigorously.
9. Centrifuge the tubes in the Heraeus Multifuge3 S-R (Thermo Scientific) at 4600 rpm for 30-45 minutes at room temperature.
10. Transfer 5 ml supernatant to a 15 ml PP tube
11. Add 1.25 ml 10 M LiCl, mix and incubate overnight at 4°C.
12. Pre-cool the Heraeus Multifuge3 S-R (Thermo Scientific) to 4°C
13. Centrifuge the tubes in the Heraeus Multifuge3 S-R (Thermo Scientific) at 4600 rpm for 45 minutes at 4°C.
14. Discard the supernatant and keep tubes standing upside down on a clean paper towel
15. Dissolve the pellet with 250 µl SSTE, pipet up and down and transfer the solution to a 1,5 ml Eppendorf tube.
16. Add 250 µl Chloroform: IAA and mix vigorously.
17. Centrifuge in a table top centrifuge for 10 minutes at 17000g (13500 rpm).
18. Carefully transfer 200 µl of the supernatant to a new tube and add 400 µl of 96% ethanol.
19. Incubate at least 2 hours at -20° C to precipitate the RNA.
20. Centrifuge for 20 minutes at 4 °C at 17.000g (13500 rpm) in a table top centrifuge.
21. Remove the ethanol and wash the pellet with 500 µl of 70% ethanol.
22. Centrifuge for 5 minutes at 4 °C at 17.000g (13500 rpm) in a table top centrifuge.
23. Discard the ethanol, centrifuge shortly and remove the remaining traces of ethanol with a small pipet.
24. Air dry the pellet for 10 to 15 minutes.
25. Dissolve the pellet in 50 µl RNAse free water; incubate for 15 minutes at 56°C in a water bath to completely dissolve the RNA.
26. Store the RNA at -80°C.
